# Role of Zinc in Immune System and Anti-Cancer Defense Mechanisms

**DOI:** 10.3390/nu11102273

**Published:** 2019-09-22

**Authors:** Dorota Skrajnowska, Barbara Bobrowska-Korczak

**Affiliations:** Department of Bromatology, Medical University of Warsaw, Banacha 1, 02-097 Warsaw, Poland; dorota.skrajnowska@wum.edu.pl

**Keywords:** zinc, immune system, cancer, defense mechanisms

## Abstract

The human body cannot store zinc reserves, so a deficiency can arise relatively quickly, e.g., through an improper diet. Severe zinc deficiency is rare, but mild deficiencies are common around the world. Many epidemiological studies have shown a relationship between the zinc content in the diet and the risk of cancer. The anti-cancer effect of zinc is most often associated with its antioxidant properties. However, this is just one of many possibilities, including the influence of zinc on the immune system, transcription factors, cell differentiation and proliferation, DNA and RNA synthesis and repair, enzyme activation or inhibition, the regulation of cellular signaling, and the stabilization of the cell structure and membranes. This study presents selected issues regarding the current knowledge of anti-cancer mechanisms involving this element.

## 1. Introduction

Cancer cells are characterized by uncontrolled division. Thus paradoxically, by striving for immortality, they end the life of a human being much faster than the passage of time. The search for new natural or synthetic compounds with anti-cancer activity is ongoing [1,2,3,4]. It is particularly important that their effect should be based on the selective pathophysiological mechanisms that have been observed in tumorigenic cells, and should not disrupt the biological balance of the patient. On this assumption, the patient’s own immune system should primarily be maximally stimulated to generate an immune response against cancerous changes. It should be noted that two-thirds of tumors are associated with human behavior: addictions, diet, a lack of exercise, excessive sunbathing, or infections. This is evidenced by marked differences in the effectiveness of treatment of different patients with the same diagnosis, and confirms that oncological treatment cannot be universal. It should also be taken into account that in the face of cancer, in addition to various forms of therapy prescribed by a doctor, patients additionally use a variety of widely available dietary supplements to help the body fight the disease, e.g., by activating the immune system. Among minerals, these will unquestionably include zinc, which performs catalytic, structural, and regulatory functions in the body, making it indispensable for the proper functioning of the body [5,6,7]. Zinc is associated with many metalloenzymes: cytoplasmic enzymes, e.g., superoxide dismutase and phosphodiesterase; mitochondrial enzymes, e.g., cytochrome oxidase and pyruvate carboxylase; nuclear enzymes, e.g., DNA and RNA polymerase; and enzymes of the Golgi apparatus, e.g., peptidase and mannosidase [7].

Zinc ions are also components of structural and regulatory proteins, including transcription factors, and form ‘zinc fingers’ (sequences enabling the binding of transcription factors to DNA) [8,9]. Zinc ions are permanently bound in these biological systems, and thus form a stable pool involved only in the specific functions of the proteins in which they are present. At the same time, zinc deficiency is a global health problem, affecting over two billion people worldwide [10,11]. In more than 10% of the population, intake of zinc with meals is less than half the recommended dose, and chronic zinc deficiencies significantly increase the risk of cancer [10,12,13]. Many patients with cancer, especially of the lungs, breast, head, and neck, have a decreased level of zinc in the blood [14,15,16,17,18,19,20,21,22]. Due to the multitude of functions performed by this element, it can be assumed to play a leading role in defending against the initiation and promotion of tumors, although the mechanism of this role is not fully known [23].

In this study, we present the mechanisms in which zinc is most frequently mentioned in scientific publications as an anti-cancer factor.

## 2. Zinc and the Immune System

In the case of anti-cancer activity, the role of zinc in the cellular and humoral immune response—the main human defense mechanism—is of particular importance [24,25,26]. A lack of or inadequate immune response to cancer cells appearing in the body may be caused by what is known as escape from immune surveillance. This is in part because the tumor grows faster than the defenses develop, and also because cytotoxic substances are blocked by various substances secreted by the tumor, e.g., transforming growth factor β (TGF-β), interleukin 10 (IL-10), also known as human cytokine synthesis inhibitory factor (CSIF), and prostaglandin E2 (PGE2) [27,28,29,30]. Due to the increased activity of TGF-β in advanced cancer, factors are sought to inhibit it. For example, the expression of TGF-β can be weakened by the administration of cytokines with antagonistic activity against this cytokine, e.g., hepatocyte growth factor (HGF), TGF-β-neutralizing antibodies, or natural inhibitors such as decorin [27,28]. IL-10 is an anti-inflammatory cytokine that inhibits the production of pro-inflammatory cytokines, such as interferon γ (IFN-γ), interleukins 2 and 3 (IL-2, IL-3), tumor necrosis factor alpha (TNF-α), or granulocyte-macrophage colony-stimulating factor (GM-CSF). The cells of certain cancers, e.g., kidney cancer, are also able to stimulate the secretion of significant amounts of the immunosuppressive mediator PGE-2 by PBMCs (peripheral blood mononuclear cells), so that the tumor causes a change in the cytokine production profile in favor of a response conditioned by Th2 lymphocytes [29]. PGE-2 inhibits the secretion of interleukin-2 (IL-2) by Th1 cells, which perform functions associated with the stimulation of an immune response, thereby modulating immune function [30]. The mechanism is most likely partly responsible for immune dysfunction in the patient, and allows cancer cells to avoid initiation of the immune response, so that the tumor grows and develops in the body ‘unnoticed’. To what extent do zinc ions influence proper immune function when mutated cells appear?

We will begin with a brief discussion of the individual elements of the immune response. The immune system includes the thymus (development and selection of T lymphocytes) and bone marrow (maturation of B lymphocytes) [31]. In these organs, as a result of negative selection, 90% of T and B cells die during differentiation, and the remaining 10% are immunocompetent lymphocytes. In addition, the immune system includes the spleen (recognition of antigens from the blood), lymph nodes (recognition of antigens in lymph), and various lymphatic tissues associated with mucous membranes, such as mucosa-associated lymphatic tissue (MALT), skin-associated lymphatic tissue (SALT), gut-associated lymphatic tissue (GALT), nasal-associated lymphatic tissue (NALT), and bronchus-associated lymphatic tissue (BALT) [32,33].

The primary function of the immune system is surveillance over mutated, damaged, and old cells that can lead to the development of cancer and autoimmune diseases. The body has a powerful arsenal to effectively destroy cancer cells, consisting primarily of natural killer cells (NK), Tc lymphocytes (cytotoxic lymphocytes), macrophages, granulocytes, cytokines secreted by immunocompetent cells, and antibodies. These elements are included in the immune response [31]. We distinguish two main mechanisms of this response: non-specific and specific. The non-specific response works when the continuity of external protective barriers has been broken, and consists in activation of the following: macrophages, granulocytes (neutrophils, eosinophils, and basophils), natural killer (NK) cells, dendritic cells, and the complementary system (they function via a mechanism of specific and non-specific immunity) [31,34]. The specific response involves B and T lymphocytes, whose activity is directed against antigens specific to particular microbial species. These include the following: B lymphocytes, which produce specific antibodies, i.e., immunoglobulin A (IgA), immunoglobulin G (IgG), immunoglobulin M (IgM) and immunoglobulin D (IgD). B lymphocytes bind free antigens, differentiate into plasma cells that synthesize immunoglobulins, and present antigens to T lymphocytes to obtain the aid of T helper (Th) lymphocytes in antibody production. The binding of an antibody to an antigen means its destruction [31,35]. T helper (Th) lymphocytes, which facilitate the activation, proliferation, and differentiation of B lymphocytes, are precursors of cytotoxic T lymphocytes (Tc), stimulate macrophages, and secrete cytokines [31]. This group includes Th17 cells, which display anti-inflammatory and anti-cancer resistance [31,36]. Tc lymphocytes exert a direct cytotoxic effect on cells infected with an intracellular pathogen. They distinguish and kill potentially cancerous cells. These include Tyδ cells (Tyδ lymphocytes), which have a cytotoxic effect on cancer cells [31,37]. T regulatory (Treg) cells suppress immune cells by secreting cytokines. In particular, lymphocytes designated with the symbol iTR are active in cancer [31,37,38]. ILC—innate lymphoid cells—are a newly described family of immune cells that are part of the natural immunity that is important not only during infections caused by microorganisms, but also in the formation of lymphoid tissue, tissue remodeling after damage due to injury, and homeostasis tissue stromal cells [39]. Family ILC cells form NK cells and lymphoid tissue inducer T cells (LTi), ILC 22, ILC 17, and ILC 2. The fifth population of ILC cells includes natural helper type 2 T cells (nTh2), innate type 2 helper cells (Ih2), multi-potent progenitor type 2 cells (MPPtype2) and nuocytes [39,40].

The immune system is particularly sensitive to changes in the level of zinc; in fact, it seems that every response is in some way directly or indirectly related to zinc. A cytotoxic effect is known to result from the secretion into the environment of cytokines, free radicals, and enzymes and substances damaging cell membranes. Cytokines produced by lymphocytes play a key role in anti-cancer immunity [41]. Cytokines are synthesized and released in a cascade: an inducing stimulus triggers the release of several cytokines that induce the expression of receptors and the synthesis of more cytokines in other cells, resulting in a coordinated response of various cell types to the original stimulus. These cytokines include the aforementioned interleukins, interferons, tumor necrosis factors (TNF-alpha and beta), and growth factors (GM-CSF) [34,35,42]. Changes in the level of zinc in the body can act as such a stimulus and interfere with both specific and non-specific immunity in various ways. Zinc deficiency in vitro impairs granulocyte recruitment, reactive oxygen species (ROS) generation, chemotaxis, and phagocytosis [43,44]. Following phagocytosis, pathogens are destroyed by active NADPH (nicotinamide adenine dinucleotide phosphate), which is also dependent on zinc (inhibited in both deficiencies and surpluses of zinc) [45,46]. The process of the adhesion of monocytes to epithelial cells is also dependent on zinc [47]. At a concentration of 500 μmol/L, zinc directly induces the chemotactic activity of leukocytes [38,48]. In vivo, low serum levels of zinc reduce the number of granulocytes and NK cells and the phagocytic capacity of macrophages [49]. Zinc deficiency increases the production of pro-inflammatory cytokines IL-1β, IL-6, and TNF-α [50]. This element is also essential for the recognition of MCH-1 (a set of proteins responsible for antigen presentation to T lymphocytes) on target cells by p58 NK receptors, and thus NK cell cytotoxicity is inhibited in zinc deficiency [37,38,51].

However, zinc exerts its strongest effect on the immune system through Th lymphocytes. Th lymphocytes include Th1 and Th2 cells. Reduced zinc content in the cell disturbs the balance between Th1 and Th2 toward Th2. The correct balance must be maintained for a proper immune response, because Th1 and Th2 lymphocytes perform different functions (immunity against intracellular versus extracellular pathogens) [42,52]. Zinc supplementation eliminates this imbalance, significantly increasing IFN-γ released from PBMCs (peripheral blood mononuclear cells), as IFN-γ is the main Th1-inducing factor. IFN-γ has antiviral, immunoregulatory and anti-cancer properties [53]. Th1 profile cytokines, i.e., IL-2, IL-12, IL-18, IFN-γ, and TNF-α, play the most important role in antitumor defense [54]. The best-known effects of IL-12 include enhancing NK cell activity, shifting the Th1/Th2 balance in favor of Th1 clones by inducing the differentiation of undifferentiated CD4 T cells toward Th1, and inducing IFN-γ secretion by Th1 and NK cells [25,54]. The increase in interferon production negatively affects the activity of cells with a Th2 phenotype and decreases the amount of cytokines release.

Th2 lymphocytes work together with Th1 cells in anti-cancer defense, supporting antibody synthesis by B lymphocytes through IL-4, IL-5, and IL-6. Th1 and Tc cells activate macrophages through IFN-γ and activate NK cells through IL-2. In addition, Th1 cells kill tumor cells directly (TNF-β) and inhibit their proliferation (interferons, TNF-β) [55].

As mentioned above, zinc exerts the strongest effect on the immune system through T lymphocytes. T-cell maturation takes place in the thymus and is dependent on thymulin, which is a peptide hormone secreted by thymic endothelial cells. In addition, zinc conditions the morphological and physiological integrity of the thymus. It acts as a cofactor in the formation of active thymulin (ZnFTS) released by thymic cells [56]. Thymulin regulates the differentiation of maturing T cells in the thymus and the function of maturing T cells in the peripheral blood. Zinc deficiency is also responsible for thymus atrophy and the consequent disruption of lymphocyte development. Minor changes in the serum zinc concentration reduce the level of T cells [12,57,58]. Moreover, thymulin induces the expression of IL-2 receptors and modulates cytokine production by PBMCs, inducing CD8 cell proliferation in cooperation with IL-2 [59].

Hence, zinc deficiency results in a decrease in the number of T and B lymphocytes in both the thymus and the bone marrow, which leads to increased susceptibility to infection and weakening of the body’s defenses [56,60,61]. As early as 1979, a significant inhibition of T-cell production was shown in zinc-deficient mice, which was reversed following zinc supplementation [61]. Zinc increases the adhesion of leukocytes to the endothelium, and the chelation of zinc ions markedly decreases their activation [60]. Zinc also stimulates the cytolytic activity of T cells [56]. Their basic task is to move through the body in search of pathogens and cancer cells, and to eliminate them without damaging healthy cells. Cytotoxic lymphocytes recognize a cancer cell through the interaction of TCR (an antigen-recognizing receptor on the T cell) with a specific oligopeptide presented by the target cell with the aid of a major histocompatibility complex (MHC) molecule. Recognition by NK cells, on the other hand, is mediated by MHC-binding receptors, while NK cells recognize cancer cells through the receptor for the Fc fragment of IgG (Fc gamma RIII; CD16) [62]. After recognition of the cancer cell, the next stage can be divided into two main types: the first is associated with the release of the contents of cytolytic granules, and the second is associated with the activation of specific receptors for molecules of the TNF superfamily. Secreted cytolytic granules (influenced by T lymphocytes) create a channel in the membrane of the attacked cell, through which sodium ions (Na^+^) penetrate it and thereby cause its apoptosis—the process of natural death [62,63]. The killing of a target cell involving cytolytic granules is believed to be more important than cytotoxic T-cell responses based on other mechanisms [62,64]. B lymphocytes are less sensitive to zinc deficiency, but it may reduce their numbers, which is probably due to the induction of apoptosis [62,64].

Many studies have linked the serum concentration of Zn^2+^ ions with the concentration of certain cytokines. However, some studies have shown that zinc supplementation increases the secretion of IL-6, IL-1β, and TNF-α, while others have not confirmed this relationship, or have even found an inverse correlation [33,64,65]. Pro-inflammatory cytokines, by acting on immune cells, take part in the mechanism of the induction of apoptosis of cells threatening the body. It is worth noting that the induction of apoptosis of peripheral blood mononuclear cells occurs only at pharmacological concentrations of zinc, i.e., levels that cannot be achieved by conventional food intake [33,64]. In a study on the effect of zinc ions on T lymphocytes, B lymphocytes, and monocytes, Driessen and colleagues [63] observed an increase in the serum concentration of certain cytokines following incubation with ZnSO_4_. Stimulation was fastest in the case of tumor necrosis factor (TNF-α), whose maximum concentration was observed after just 16 h [63]. After 24 h, interleukin-1β (IL-1β) and interleukin-6 (IL-6) reached their maximum concentrations. All of these cytokines are produced by monocytes; therefore, the authors concluded that this type of leukocyte is most strongly stimulated by zinc ions. Interferon γ (IFN-γ) produced by T cells reached its maximum serum level much later—not until the sixth day. The results suggest that the increased production of T lymphocytes in the case of zinc ions is a secondary response (an effect of the cytokine cascade), and that it is interleukin-1β (IL-1β) produced by monocytes that is responsible for their stimulation [63,66].

Zinc supplementation in the amount of 20 mg/day zinc for five weeks in children with zinc deficiency was found to increase the percentage of CD4+ and CD8+ cells, and in older individuals, 48-day supplementation led to an increase in Th lymphocytes [67].

Other research [68] has shown that zinc supplementation (5 mg/kg) for four weeks significantly increased the number of NK cells. NK cells play a key role not only in the direct killing of target cells, but also in sending signals that stimulate an immune response [68]. Thus, NK cells are involved in processes preventing cancer, and zinc is necessary for their activation [34,67].

Anti-cancer activity has been demonstrated in particular in the case of Th17 helper cells [69], cytotoxic lymphocytes (Tc) with the NK symbol [68], and Treg regulatory lymphocytes (iTr) [70]. The means by which Th17 cells modulate the immune response has not been determined. Thus far, no direct influence of Th17 lymphocytes on either tumor cells or the cytotoxic properties of T lymphocytes has been demonstrated. Therefore, they are believed to affect the anti-cancer response indirectly by increasing the expression of co-stimulatory molecules and MHC class II molecules on dendritic cells (thereby accelerating their maturation); by attracting cytotoxic T lymphocytes and NK cells to the tumor environment; and by inducing the production of chemokines, such as CXCL9 and CXCL10 [69]. IL-17, as a pleiotropic cytokine, may also stimulate tumor growth, as it has been shown to promote the growth of cancer cells through pro-inflammatory and proangiogenic activity. Zn has been shown to inhibit Th 17-mediated autoimmune diseases, partially inhibiting their development by impairing the activation of signal transducer and activator of transcription 3 (STAT3). In mice injected with type II collagen to induce arthritis, treatment with Zn inhibited the development of Th17 cells. STAT3-mediated activation of IL-6 and the development of Th17 cells in vitro were inhibited by Zn. Importantly, Zn binding altered the alpha-helical secondary structure of STAT3, disrupting the association of STAT3 with JAK2 (Janus kinase-2) kinase and with a phosphopeptide that contained a STAT3-binding motif from the IL-6 signal transducer gp130. The final conclusion was that Zn suppresses the activation of STAT3, which is an important step in Th17 development [71]. At the same time, there is a great deal of data supporting the anti-cancer effect of Th17 lymphocytes. It seems to be largely dependent on the advancement of the disease (having a different role in the early and late stages), as well as the origin of the cancer and the role of inflammatory processes and angiogenesis in its pathogenesis. The immunogenicity of the tumor is also significant, as the inhibition of tumor cell growth by Th17 lymphocytes has been demonstrated only in immunogenic tumors [72].

Zinc is not as important for the development of B lymphocytes as for T lymphocytes. Low levels of zinc in the body lead to a reduction in the total number of B cells and their precursors as well as antibody production. However, the changes in maturing B-cells are insignificant. The changes in the number of B cells are likely caused by the induction of apoptosis [70]. Glucocorticoids secreted in response to zinc deficiency provoke increased apoptosis in immature B and T lymphocytes in the bone matrix and thymus [73].

**Figure 1:**
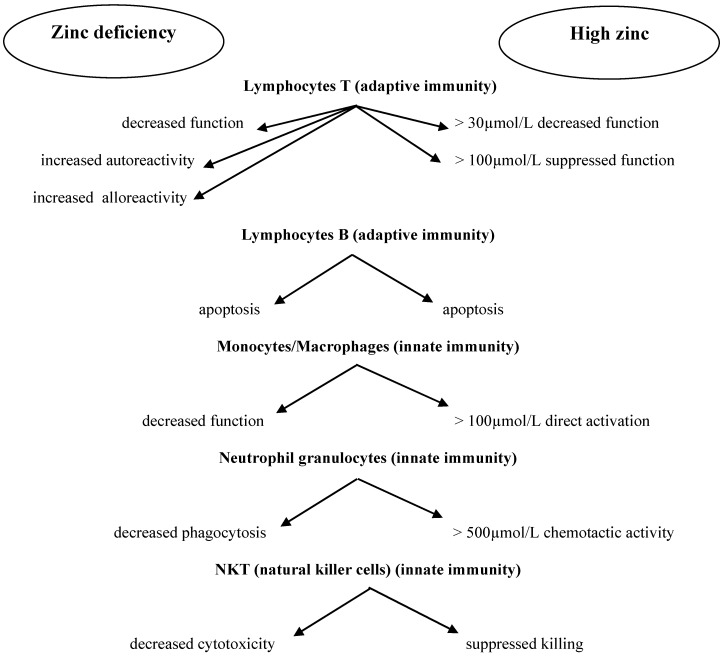
Correlation between zinc concentration and cells of adaptive and innate immunity.

In addition to the positive effect of zinc on human immune cells, an unfavorable phenomenon has been observed as well: in some situations, Zn^2+^ ions promote the multiplication of pathogens [74]. Excess zinc can also be dangerous due to its immunosuppressive effect. In high doses, it may exert an immunosuppressive effect by inhibiting lymphocyte function and IFN-γ production [56,59,75]. This immunosuppressive effect of zinc may have a new therapeutic application in autoimmune diseases, such as rheumatoid arthritis or graft-versus-host disease, in which the selective suppression of lymphocyte function is beneficial [76,77]. Figure 1 presents the correlations between zinc concentration and immune cell function [78]

It is worth noting that under certain conditions, an intake of zinc during the course of anti-cancer treatment may be harmful. The chemotherapeutics used may interact with elements taken as supplements, so all preparations taken by the patient should always be taken into account [77].

To sum up, numerous studies have shown that zinc deficiency reduces monocyte adhesion to the endothelium, granulocyte chemotaxis, macrophage phagocytosis, the activity of cytokines secreted by T cells and macrophages, NK activity, T-cell differentiation, and the release of certain interleukins and antibodies. These are the immune system’s main mechanisms of anti-cancer activity.

### Human Intestinal Microbiome and Immune System

It is worth noting that the human intestinal microbiome significantly affects the functioning of the immune system. The colonization with commensal bacteria leads to the differentiation of naive lymphocytes (Tn) into regulatory lymphocytes (Treg) and to the production of IL-10, the development of immune tolerance, the maintenance of cytokine balance, and food intolerance. The state of dysbiosis is associated first of all with the induction of certain gastrointestinal disorders, as well as obesity, diabetes, allergy, depression, or autism [79,80]. Numerous studies also prove that there is a correlation between commensal microbiome and cancer [81,82,83,84,85,86,87,88,89,90]. The ways in which gut microbiota shape the immune response in the intestine and on the perimeter are not yet fully known. It seems that only certain groups of microorganisms differing in immunomodulatory properties and the proportions between them take part in the system of steering the immune response. In the hitherto-performed investigations, several bacteria have been identified as being responsible for the immune regulation within the intestine, among other things via the regulation of the ratios of Th1/Th17/Th2 and Th1/Th17/Treg [91,92].

It has been emphasized recently in multiple articles that gut microbiota exert a very significant effect on the immune checkpoint blockade [87,88,90,93].

As it was mentioned earlier, specific immunity involves the cells and their receptors, which are responsible for the specialized identification of foreign antigens and the reaction to danger, i.e., both the external pathogens and self cells of malformed structure or function, such as cancer cells. The identification of foreign antigens occurs as a result of the presentation of fragments of those antigens complexed with the major histocompatibility complex (MHC) molecules, to specific antigen receptors of T lymphocytes known as T-cell receptors (TCR). Besides, in terms of close control of the specific immune response, the T-cell receptors receive a set of co-stimulating or inhibiting signals that are provided by special receptors and their ligands, which are known as the “immune checkpoints” [92,94,95]. This makes it possible to avoid the response against self-antigens, which are present in healthy cells. Thanks to the immune checkpoints, the immune system remains in the state of balance, simultaneously eliminating the pathogens and maintaining tolerance for self-antigens. A too-strong immune response to the pathogens or the alteration of self-antigens can result in the cell damage and cause autoimmune diseases.

The T-cell receptors recognize the altered antigens (called neoantigens) present on the surface of cancer cells, which leads to the damage of cancer cells and tumor growth inhibition. It has been proved that the immune response associated with T lymphocytes affects the patients’ survival and can be a prognostic factor for the response to immunotherapy [94].

However, at a certain moment, the “exhaustion” of immune system cells can occur, including T-cell lymphocytes. In such a case, first of all, the “exhausted” T lymphocytes exhibit a lower ability of proliferation and cytokin production, and secondly, there appears to be an increased expression of the receptors that inhibit the immune response, which are known as the immune checkpoints. These receptors are bound to corresponding ligands that are present on the surface of cancer cells and other cells in the tumor microenvironment. Using those checkpoints, the tumor cells are able to block the anti-cancer T lymphocytes, as a result of which cancer cells can escape the surveillance of the immune system [95].

It seems that zinc can strongly influence the gastrointestinal microbiome due to its powerful effect on various mechanisms of the immune system and thus on the immune response, and also by affecting the permeability of the epithelium against the attacking pathogens and microorganisms or reducing mucosal inflammation of the gastrointestinal mucosa [96,97]. In developing countries such as Bangladesh, zinc is an inexpensive and affordable strategy to overcome diarrhea [98,99,100].

However, it is worth emphasizing that each person has an entirely unique microbiome that reacts differently to both internal and external factors. Excessive zinc supplementation can adversely affect the microbiome structure and increase the risk of infection, e.g., with Clostridium difficile [101]. These bacteria made the symptoms of the disease worse and also caused a higher mortality rate in mice that were supplemented with excess amounts of zinc. This was probably due to the increased activity of toxins and the change of host immune response [101].

## 3. Zinc and Nuclear Transcription Factor Nf Kappa B (Nf-κB)

The initiation, promotion, and progression of tumors involves various signaling pathways with the participation of transcription factors such as nuclear factor E2-related factor 2 (Nrf2) (induction), NF-κB (inhibition), and activator protein-1 (AP-1) (inhibition). A number of chemopreventive agents can induce or inhibit their action. Zinc influences gene expression at the level of the cell nucleus by stabilizing the structure and regulating various transcription factors, including NF-κB.

NF-κB in its active form induces the expression of about 200 genes associated with angiogenesis, metastasis, cell proliferation [36,78,102,103], resistance to certain chemotherapeutics [24,42,78], and the ability to inhibit tumor cell apoptosis, and also promotes tumor formation [78,104,105]. In several types of cancer, e.g., chronic lymphocytic leukemia, melanoma, and pancreatic, breast, prostate, colon, bladder, and lung cancer [24,42], a permanent constitutive activity of NF-κB has been observed [106]. This factor is present in the cytoplasm as an inactive complex that is bound non-covalently to the inhibitory proteins known as IκBs (α, β, γ and c) [78]. Its inhibitory subunit IκB prevents the migration of NF-κB to the cell nucleus. The signal cascade is activated by the classic route by the liposaccharide (LPS) in the envelope of Gram-negative bacteria, viruses, or pro-inflammatory cytokines. Then, the IκB subunit is phosphorylated by I kappa B kinase (IKK) kinases and subsequently recognized by the Skp1p–cullin–F-box protein (SCF) ubiquitin–ligase complex, which leads to the rapid ubiquitination of IκB followed by its degradation by 26S proteasome and the release of NF-κB dimers. The active free form, which is most frequently present in the form of a dimer with p50/ReIA proteins (p50/p65), migrates with the exposed NLS (nuclear localization signal) sequence to the cell nucleus, where it binds to a specific site in the DNA and then induces the expression of target genes, particularly pro-inflammatory cytokines IL-1β and TNF-α produced by macrophages, interleukin (IL), cyclooxygenase-2 (COX-2), inducible nitric oxide synthase (iNOS), or adhesion factors [78,104,105].

Therefore, NF-κB is thought to have a role in enhancing inflammatory reactions. In many chronic inflammatory diseases, NF-κB is permanently active and supports pathogenic processes. However, gene expression controlled by transcription factors that recognize and bind DNA regulatory sequences may be modified by various biologically active compounds. Research by Riehemann et al. demonstrated the inhibition of the synthesis of cytokines regulated by the NF-κB transcription factor [107]. This creates the opportunity for early therapeutic intervention at the level of gene expression by acting on NF-κB. One of the important methods of blocking NF-kB is to prevent its activation, i.e., its transfer to the cell nucleus. As a result, NF-κB does not have access to the pro-inflammatory genes it regulates, which prevents an inflammatory reaction.

There are several strategies to block its activation: influencing the redox status in cells [108] by means of antioxidant compounds (e.g., glutathione or vitamin C) [109]; the use of competitive inhibitors that bind to the NF-κB site in the DNA chain, e.g., elements (zinc, chromium, cadmium, or gold) or peptides such as transcription factor decoy (TFD), IL-4 interleukin, or vascular endothelial growth factor (VEGF) [109]; or the use of inhibitors of the 26S proteasome, which is needed for degradation of the NF-κB–IκB complex, such as cyclosporine A, lactacystin, and proteasome inhibitor PS-341 [108,109,110]. The last case involves exploitation of the fact that cancerous cells, which are genetically unstable, synthesize large quantities of abnormal proteins. Blocking the breakdown of such proteins by inhibiting proteasomes causes them to accumulate in the lumen of the endoplasmic reticulum, leading to caspase activation and cell death. Therefore, compounds that inhibit proteasome activity are currently used in cancer therapy [111]. Other ways to block NF-κB activity include the use of inhibitors of phosphorylation of IkBcx, which belongs to the inhibitory proteins of NF-kB (IkB) family (e.g., prostaglandin A1 or nitric oxide) [109,110,111,112]; the use of compounds blocking the transport of an active dimer to the nucleus [109]; the introduction into cells of genes coding for modified suppressor proteins with mutations at sites specific for phosphorylation and ubiquitination [109,113]; and finally, the introduction of the gene encoding histone deacetylase (HDAC3), which directly interacts with the p50/RelA protein, enabling effective binding of the newly synthesized protein IicBa to the p65/p50 dimer and expulsion of the NF-kB complex from the nucleus [109,114].

In the case of the effect of zinc on the NF-κB transcription factor, the scientific literature contains reports of both inhibition and induction, in a variety of indirect mechanisms. In vitro studies by Haase et al. [38,115] found that zinc is necessary for the activation of lipopolysaccharide (LPS) inducing the NF-kB signaling pathway, and the use of the zinc chelator TPEN (N,N,N0,N0-tetrakis-(2-pyridylmethyl) ethylenediamine) stopped the induction of the NF-kB pathway [67]. On the other hand, it is emphasized that zinc can negatively regulate the NF-kB signaling pathways through numerous mechanisms [116].

Several NF-κB-inhibiting mechanisms associated with the presence of zinc ions have been described:

### 3.1. Zinc and Cyclic Nucleotide Phosphodiesterases (PDEs)

Zinc modulates the activity of NF-κB by acting as a reversible inhibitor of cyclic nucleotide phosphodiesterase (PDE) [117]. This is because PDE catalyzes the hydrolysis of the 3’-phosphodiester bond in cyclic nucleotides, resulting in the formation of nucleoside-5’-phosphate [118,119]. Increased concentration of cAMP (adenosine 3’,5’-cyclic monophosphate) and cGMP (cyclic guanosine monophosphate) in the cell leads to the cross-activation of protein kinase A (PKA) and inhibition of the phosphorylation of protein kinase Raf-1 (RAF proto-oncogene serine/threonine-protein kinase) [120]. The phosphorylated residue Ser259 in the CR2 domain of the Raf protein probably acts as a suppressor of this protein’s activity, and via feedback mechanisms cause conformational changes that reduce the affinity of the CR3 domain for kinases and inhibit the phosphorylation cascade of the MAPK (mitogen-activated protein kinases) signaling pathway [121,122,123].

Therefore, cyclic AMP may inhibit the expression of pro-inflammatory cytokines, e.g., by the NF-κB transcription factor [124]. There are reports indicating a correlation between the inhibition of PDE activity and the suppression of TNF-α production following the LPS stimulation of mononuclear cells obtained from the blood of healthy volunteers. This study showed a linear relationship between half-maximal inhibitory concentrations (IC50) for the inhibition of PDE and TNF-α in the presence of PDE inhibitors, 3,7-dimethylxanthine derivatives, and selected 3-methylxanthine derivatives [125]. Zinc has also been shown to suppress the LPS-induced activation of IκB inhibitor kinase-β (IKKb) and NF-κB, and subsequent TNF-α production in human monocytes [105,126,127].

The cAMP-dependent protein kinase A (PKA) signaling pathway is known to play a major role in many pathophysiological conditions, and although there is conflicting evidence concerning the effect of cAMP/PKA on nuclear factor NF-κB, most studies confirm that an increase in intracellular cAMP and/or PKA activation negatively modulates NF-κB transcriptional activity. In addition, the inhibitory effect of the cAMP/PKA pathway on the transcriptional activity of NF-κB is manifested as a modification of the C-terminal transactivation domain of p65, directly or indirectly [128].

Similarly, zinc can bind to a zinc finger-like motif found on protein kinase C (PKC) and inhibit the PMA (phorbol 12-myristate 13-acetate)-mediated translocation of PKC to the membrane. When this occurs in mast cells, NF-κB activity is indirectly inhibited [129]

### 3.2. Zinc and Zinc Transporters

Zn homeostasis within cells is maintained by the ZIP (zinc-iron permease) and ZnT (zinc transporter) protein families and by metallothioneins. ZIP family proteins are involved in the influx of Zn ions into the cytosol from outside the cell or from intracellular vesicles, while ZNT proteins transport zinc in the opposite direction. Zinc can affect NF-κB through its transporter ZIP8 [130,131]. Zinc transporter ZIP8 (SLC39A8) is a transcriptional target of NF-κB and an important response to cytokines, bacteria, and infection. ZIP8 increases zinc uptake from the extracellular environment or its release from intercellular organelles, thereby increasing the zinc content in the cytosol. The increased level of zinc induces the inhibition of NF-κB (activation of the MAPK cascade and blocking of the NF-κB–IKK complex). Therefore, ZIP8 is a regulator of the NF-κB negative feedback system, acting via the zinc-mediated inhibition of IKK in response to infection [130,131]. It is worth noting that zinc transporters from the ZIP family, although they perform a seemingly similar function, have thus far shown varied effects on the progression of some cancers in research. Some of them are associated with the progression of cancer; for example, membrane transporter ZIP10 promotes the metastasis of breast cancer cells to the lymph nodes. The use of a zinc-chelating compound has been shown to inhibit the migration of breast cancer cells of the invasive MDA-MB-231 line [132]. Similar results were not found after the chelation of copper and iron. However, only Zn transported by ZIP10, and not free Zn ions in the cytosol, was found to be necessary for the migration of breast cancer cells [132]. An increase in ZIP7 expression, on the other hand, is associated with the resistance of breast cancer cells to cancer treatment and an increase in their growth and invasiveness [133], while an increased level of another zinc transport protein, ZIP6, has been associated with a better prognosis and longer survival of patients with breast cancer [134].

### 3.3. Zinc and Expression of Protein A20

One of the inhibitors of NF-κB is protein A-20, whose expression is induced by zinc. Zinc is part of the structure of this protein (as a zinc finger domain), and induces its expression [135]. Protein A20 consists of 790 amino acids. The C-terminal domain has a zinc finger structure, and the N-terminal domain is a characteristic of the cysteine protease superfamily [136]. The N-terminal domain contains deubiquitinating enzyme motifs (DUBs) [137], but A20 generally does not perform the function associated with DUBs [138]. The main mechanism of action is based on the C-terminal domain consisting of seven zinc fingers. The fourth zinc finger conditions ubiquitin ligase activity, and the rest of the C-terminus is responsible for integration with lysosomes and the degradation of signaling molecules [135,139]. Protein A20 degrades a number of proteins or deactivates them by binding with them, preventing activation of the NF-κB pathway. In pathways initiated by pathways of toll-like receptors (TLRs) and tumor necrosis factor receptors (TNFRs) (the induction of pro-inflammatory cytokines), zinc-finger protein A20 is the main negative regulator of NF-κB activation [140]. The effect on NF-κB through the TLR2 and TLR4 receptor pathways is based on the inhibition of TNF-receptor-associated factor 6 (TRAF6) interacting with interleukin-1 receptor-associated kinase 1 (IRAK-1) to stimulate the TGFβ-activated kinase complex (TAK1)/TAK-1-binding protein 1 (TAB) and subsequently in the activation of IκB inhibitor kinases IKKα and IKKβ [23,141]. The lack of IκB phosphorylation ensures that free NF-κB will not enter the cell nucleus and activate pro-inflammatory genes. In vitro studies on mice have demonstrated that during the stimulation of this transcription factor by LPS, the A20/TRAF6 mechanism prevents the activation of NF-κB [141]. Blocking NF-κB activation by A20 also takes place in a TNF-α-dependent manner. The binding of TNF-α to its receptor results in the recruitment of the DD domains of TNFR, which then interact with RIP (receptor-interacting protein) or with TRAF2 (TNF-receptor associated factor 2) [142]. During TNFR signaling, A20 is able to deubiquitinate receptor interacting protein 1 (RIP1), which prevents its interaction with the NF-κB essential modulator IKKγ. There is no activation of IKK kinase with the subsequent degradation of the IκB inhibitor. NF-κB remains in the cytoplasm.

Additionally, zinc supplementation has been shown to downregulate inflammatory cytokines by decreasing the gene expression of IL-1 and TNF through the upregulation of mRNA- and DNA-specific binding for A20, subsequently inhibiting NF-κB activation [143].

### 3.4. Zinc and Peroxisome Proliferator-Activated Receptor α (PPAR-α)

In addition, zinc inhibits NF-κB activation at the level of DNA binding by increasing the expression of peroxisome proliferator-activated receptor α (PPAR-α), which is responsible for lipid catabolism and intracellular metabolism [144]. Activation of this receptor induces the transcription of the major enzymes involved in the β-oxidation of fatty acids, taking place in both peroxisomes and in the mitochondrion, microsomal ω-oxidation, the synthesis of ketone bodies, and the transport and transformation of fatty acids [144]. An increase in PPAR-α leads to the downregulation of inflammatory cytokines and adhesion molecules.

## 4. Zinc and Antioxidant Processes

Another mechanism by which zinc potentially inhibits tumor growth, while largely associated with its effect on the activity of the NF-κB transcription factor, is primary associated with its antioxidant properties. The intracellular redox potential control system is an indispensable element ensuring the maintenance of cellular homeostasis, as well as a regulator of many metabolic functions of cells [145]. In conditions of homeostasis, reactive oxygen species (ROS) play the role of mediators and regulators of numerous cellular processes [146]. At physiological concentrations, ROS induce differentiation and apoptosis; the synthesis, release, or inactivation of NO; and glucose transport to cells; and also regulate cellular signaling to and within the cell [147]. Moreover, by altering the permeability of capillary walls, they condition the correct course of the inflammatory reaction. Protection against excessive concentrations of ROS having toxic effects is provided by the antioxidant system. The central role in the system is played by mitochondria, which are not only the main source of production of reactive oxygen species, but also have an extensive antioxidant system [148]. The antioxidant system consists of enzymatic proteins such as superoxide dismutase (SOD 1, SOD 2), catalase (CAT), glutathione peroxidase (GPx), glutathione reductase (GR), and peroxyredoxin (PRX), as well as non-enzymatic proteins such as glutathione (GSH) and thioredoxin (Trx), vitamins C and E, and metal ions [149,150,151]. An increased or prolonged excess of ROS disturbs a number of biological functions of lipids, DNA, and proteins by permanently changing their structure, leading to abnormalities in cellular metabolism [152]. Interestingly, in tissue cultures, zinc has been shown to protect healthy cells against the cytotoxicity and genotoxicity of hydrogen peroxide, but intensifies the toxicity of H_2_O_2_ in tumor tissue [153].

### 4.1. Zinc and Antioxidant Enzymes

Zinc has potent antioxidant activity, primarily as a component of superoxide dismutase (SOD 1, SOD3), catalyzing the dismutation of superoxide anion radicals to hydrogen peroxide and thus preventing the generation of other toxic free radicals and their derivatives, e.g., hydroxyl or peroxynitrite radicals [154,155]. Given the function of SOD in the cells of the body, it seems that it may play a significant role in the pathology of cancer [156,157]. SOD is present in the body in the form of three isoenzymes: copper, zinc superoxide dismutase (CuZnSOD) found in the cytosol, manganese superoxide dismutase (MnSOD) located in the mitochondria, and extracellular superoxide dismutase (EC-SOD) present in extracellular spaces and fluids [155]. Together with copper, zinc ions constitute a prosthetic group in SOD1 and 3 that provides resistance to the chemical or physical denaturation and stabilization of the tertiary structure of the enzyme [158,159]. Metal atoms are complexed to apoenzyme by histidine residues. SOD1 activity accounts for 50%–80% of the total activity of superoxide dismutases [160]. A reduction in enzyme activity, causing oxidative stress, leads to neuron death and tumor progression [161].

CuZnSOD and MnSOD have been shown to influence the formation and development of tumors [156,157]. An increase in SOD family enzymes is induced by factors such oxidative stress, TNF-α, IL-1, and liposaccharides. Expression of the cytosolic dismutase (CuZnSOD) gene is not regulated by cytokines, but by nitric oxide [162]. The induction of CuZnSOD gene expression by nitric oxide controls the proliferation of human keratinocytes, and possibly other cells as well. A mutation in the CuZnSOD gene causing a decrease in its expression may result in an inappropriate response to nitric oxide and a loss of control over cell proliferation, which may lead to neoplastic transformation. The induction of SOD gene expression in cancer cells in response to factors inducing oxidative stress is highly varied, and seems to depend mainly on the stage of cancer [163,164]. Primary tumors typically have low activity and low levels of expression of CuZnSOD as compared to healthy tissues. As the cancer develops from the primary tumor to metastatic tumors, the activity and expression of CuZnSOD may increase. However, it should be noted that the contribution of individual SOD isoenzymes (CuZnSOD and MnSOD) to the development of neoplastic changes is varied [164,165].

The link between the deficiency of superoxide dismutase and the development of serious diseases has increased interest in these enzymes. Attempts have been made to use superoxide dismutase mimetics containing transition metal ions of varying stability and activity in the fight against free radicals and reactive oxygen species during pathological processes in living organisms [166,167]. On the other hand, already formed cancer cells produce large quantities of ROS, and so their survival is dependent on the activity of antioxidant enzymes. Therefore, it has been postulated that the suppression of antioxidant enzyme activity may result in greater oxidative damage than in normal cells and induce apoptosis. This hypothesis has been supported by the results of studies showing that cancer cells of patients with leukemia and ovarian cancer are more sensitive to the inhibition of SOD activity by 2-methoxyestradiol (2-ME) than normal cells. The mechanism of action of 2-ME consisted of inhibiting SOD activity through the accumulation of significant quantities of superoxide anion radicals. This accumulation resulted in damage to the mitochondrial membranes, the release of cytochrome c, and the activation of apoptosis in tumor cells [168,169,170,171,172].

### 4.2. Zinc as an Iron and Copper Antagonist

Another type of mechanism of the antioxidant action of zinc involves its antagonism against elements involved in lipid peroxidation (iron and copper). The zinc ion, which is inactive in this respect, displaces copper and iron from membrane-binding sites, thereby preventing them from generating free radicals. Hydrogen peroxide itself does not exert a strong oxidizing effect, but easily penetrates cell membranes, and in the presence of transition metal ions (e.g., Fe^2+^ and Cu^1+^), together with the O^2•^ radical, it can be a source of unstable but highly reactive hydroxyl radical OH^•^ and singlet oxygen (Fenton reaction, Haber–Weiss reaction) [173,174].

### 4.3. Zinc and Protein Keap1 and Transcription Factor Nrf2

Zinc also participates in the protection of protein sulfhydryl groups against oxidation through the formation of chelates, impeding spatial changes. One example is Kelch-like protein ECH-associated protein (Keap1), which has numerous cysteine residues on its surface that can be oxidized (e.g., as a result of an increased number of free radicals in the cell). The oxidation of cysteine residues results in the alteration of the structure of protein Keap1 and the release of zinc ions and factor Nrf2 from the complex [175,176].

In addition, an increase in the concentration of free zinc due to the breakdown of protein chelates with zinc (e.g., by ROS) [177] may act as a chemopreventive factor in regulating the response to oxidative stress by means of transcription factor Nrf2. Its role is to protect cells from the harmful effects of oxidative stress. Under normal conditions, Nrf2 is present in the cytoplasm in a form bound to the cytoskeleton protein Keap1. Under stress conditions, excess ROS and electrophiles cause Nrf2 to dissociate from the inactive complex with Keap1, so that it translocates to the nucleus and binds to the DNA molecule. This leads to the transcription of genes encoding cytoprotective proteins, such as phase II enzymes or low-molecular-weight antioxidant proteins (e.g., thioredoxin, ferritin, or metallothioneins) that are responsible for protecting the cell against reactive oxygen species (ROS). Both the aforementioned conformational changes in protein Keap1 and the phosphorylation of Nrf2 may underlie the dissociation of the Nrf2–Keap1 complex. In the first case, key importance is ascribed to the cysteine residues in the structure of Keap1, whose reactivity is modulated by the binding of zinc [175]. Thus, Keap1 acts as a ‘sensor’ of oxidative stress, and is a particle that is indirectly responsible for regulating the expression of Nrf2-dependent genes [176].

### 4.4. Zinc and Metallothioneins (MTs)

One of the characteristics of neoplastic transformation is the loss of cells’ capacity for apoptosis. Gene mutations leading to the development of cancer cause an impairment of cell cycle regulation mechanisms and uncontrolled division. These disturbances involve the loss of balance between pro- and anti-apoptotic factors and damage to proteins taking part in programmed cell death. An increased expression of MT (metallothionein) isoforms has been shown in some tumor cells and translated into a truncated G1 phase and a faster transition to the S and M phases of the cell cycle [178]. Zinc has been shown to increase the synthesis of metallothioneins, which are proteins rich in thiol cysteine residues (20 of 61 amino acids are cysteine), acting as free radical scavengers in skin, liver, and bone marrow cells [44,179,180]. Metallothioneins are important regulators of the level of zinc in cells. Zinc and copper are physiological inducers of metallothioneins, although they also bind other divalent ions. A single metallothionein molecule is capable of binding seven divalent zinc ions and up to 12 monovalent copper ions [181].

Metallothioneins have been identified in both the extracellular and intracellular environment. Their intracellular pool is responsible for the detoxification of heavy metals (Cd, Hg) and organic compounds and for the removal of reactive oxygen and nitrogen species. In the extracellular environment, metallothionein acts as an antioxidant, transports elements between tissues, and takes part in the cellular response to stress. We distinguish metallothionein types 1, 2, 3, and 4. The type 1 and 2 metallothionein gene is expressed in various tissues, whereas type 3 is expressed only in the brain. The expression of type 4 metallothionein is limited to the stratified squamous epithelium of the skin and the upper parts of the gastrointestinal tract [182]. The MT-1 and MT-2 isoforms participate in Zn metabolism, contributing to the preservation of its homeostasis through distribution, storage, and release [183]. They regulate absorption through competition or the support of transport via protein transporters. Zinc is released from metallothionein very quickly, most likely with the participation of transcription factor 1, which is dependent on metal ions (MTF1) [180,184,185]. MTs also influence the activity of Zn-dependent proteins, some enzymes (Cu-Zn SOD), zinc finger proteins, and transcription factors, such as specificity protein 1 (Sp1) or transcription factor IIIA (TFIIIA), as well as other proapoptotic proteins [180,186]. Their role in the carcinogenesis process, and indirectly the role of zinc, cannot be overstated. Zn ions are necessary in maintaining the stability of protein p53 and its affinity for DNA. Increased concentrations of MT-1 and MT-2 cause the removal of Zn, which leads to the destabilization and inactivation of p53, thereby inhibiting apoptosis. This mechanism has been confirmed in clinical trials in patients with colorectal cancer [187].

Numerous studies have also shown that MT induces many anti-apoptotic B-cell lymphoma 2 (Bcl-2) oncogenes and a regulatory gene coding for transcription factor c-myc, while inhibiting the activity of proapoptotic proteins, such as caspase-1 and caspase-3. The relationship between the increased concentration of MT-1 and MT-2 isoforms and reduced caspase-3 activity is explained by the fact that Zn ions are necessary for caspase-3 activity, as in the case of protein p53 [188,189].

The inverse relationship has been demonstrated between MT expression and cell susceptibility to apoptosis, and thus an anti-apoptotic effect is ascribed to it [190]. Increased MT expression has also been observed to be associated with the rate of tumor cell proliferation [191] and with increased MT expression in malignant tumor cells with respect to benign forms [192]. Molecular functions and the involvement of MT in proliferation, angiogenesis, and apoptosis indicate its important role in the development and course of carcinogenesis and the emergence of multidrug resistance (MDR) [192].

One of the pathogenic factors in cancer is inflammation, which is often characterized by increased MT expression. A strong inducer of ROS, nuclear factor kappa-B (NF-κB) described above, underlies inflammation in the cell. It has been demonstrated that MT-1 and MT-2 isoforms can inhibit its activity [109,193,194,195].

MT can stimulate a chemotactic response to inflammation by leukocytes and macrophages. The increase in MT expression in damaged tissues, especially in the first two days, may result from increased concentrations of cytokines (IL-1) and growth factors. Metallothioneins have also been shown to suppress allergic reactions, acting as antioxidants and inhibitors of cytokine release [179,188].

As mentioned above, metallothioneins not only bind divalent ions, but are also strong ROS scavengers. For example, the ability of metallothioneins to scavenge hydroxyl radicals is 300 times greater than the antioxidant capacity of glutathione. In addition, these proteins protect the DNA structure from oxidation by supplying zinc ions. Chronic zinc deficiency increases susceptibility to ROS-induced damage. Altered zinc metabolism in the course of neurodegenerative diseases (e.g., Alzheimer’s disease) is associated with metallothionein deficiency and neuron oxidation [183,184,185]. The deletion of genes encoding MT-1 and MT-2 isoforms in mice resulted in greater oxidative stress in the brain cells and higher mortality than in mice with normal expression of the genes [193]. An increased expression of MT genes made the animals resistant to oxidative stress, whereas mice with silenced MT genes were much more susceptible to ROS-induced oncogenesis [196]. Research on the anti-inflammatory activity of the MT-1 and MT-2 isoforms has demonstrated their ability to inhibit the synthesis of pro-inflammatory enzymes cyclooxygenase 2 (COX-2) and inducible nitric oxide synthase (iNOS) [197]. Metallothioneins are also involved in the angiogenesis process. Numerous in vivo and in vitro studies have demonstrated that an increased expression of the MT-1 and MT-2 isoforms may potentiate tumor angiogenesis, e.g., by increasing the synthesis and expression of fibroblast growth factor (FGF), transforming growth factor (TGF-β), and vascular endothelial factor (VEGF), leading to better blood supply to the tumor and stimulating its growth [197,198].

MT is induced as a result of many different stimuli, including the presence of metals, hormones, cytokines, oxidative stress, or inflammatory factors [181], but in most of them, the zinc ion plays an enormous role. Some functions of MT, such as its antioxidant, regenerative, angiogenic, and detoxifying activity can contribute to the development of cancer, while its anti-inflammatory activity can suppress cancer. However, based on the available data, it cannot be determined conclusively whether increased MT expression only performs the role of a factor stimulating the initiation and development of tumorigenesis or whether it is a factor that inhibits the development of a tumor or its malignant transformation.

## 5. Zinc and Zinc Fingers

Transcription factors are a diverse group of proteins that regulate transcription processes, both as repressors and activators, binding to DNA through certain characteristic structural motifs [199]. The main transcription factors distinguished are zinc fingers, nuclear receptors (both motifs are stabilized by four coordinated zinc ions), and leucine zippers (stabilization through hydrophobic interactions between numerous leucines). Proteins containing zinc fingers, owing to their highly selective ability to bind to DNA, RNA, or other proteins, can perform a variety of functions [200,201,202]. Eight types of zinc fingers are distinguished, according to the quaternary structure and the location of the amino acids coordinating zinc [8]. The best known is the C_2_H_2_ family, in which the zinc ligands are two cysteines and two histidines, and the structural motif includes an α-helix and two short antiparallel β-sheets.

Knowledge of the mechanism of binding of zinc fingers to DNA can be exploited to design proteins that bind to a precisely defined DNA sequence [203]. During binding, the protein domain attains a configuration reminiscent of fingers gripping a rod [204]. Combined with the nuclease domain, zinc fingers designed in this manner create fusion proteins (zinc finger nuclease, or ZFN) that can be used in gene therapy [205]. This allows a correct copy of the inoperative gene to be inserted into the cell or enables the disruption of oncogenes. There were high expectations associated with the production of proteins with zinc finger nucleases (ZFN), which is called zinc finger nuclease-based engineering, and could be applied both in biotechnology and in medicine. However, those expectations have been tempered recently when it occurred that ZFNs are characterized by considerable cytotoxity, which in most cases is associated with the phenomenon of unfavorable hydrolysis of sequences in the genome other than the targeted one—although similar to it—called off-target sequences [206]. A better alternative occurred to be the CRISPR–Cas system (clustered regularly interspaced short palindromic repeats-CRISPR-associated) in which the short complementary RNA molecule [207] (and not the protein as in the case of ZFN) is responsible for the identification of DNA. Therefore, better understanding of the interactions of zinc fingers and nucleic acid is essential.

In conclusion, zinc performs very important functions in the cell by stabilizing zinc finger structures, playing an important role in the regulation of DNA replication and repair, transcription and translation, cell proliferation and maturation, and apoptosis [8,44].

### 5.1. The Effect of Zinc on Genes

Zinc ions can bind to a specific membrane receptor, initiating a signal transduction cascade, and stabilizing biological membranes by contributing to the fluidity of the lipid layer. At the level of the cell nucleus, zinc can induce gene expression through the structural stabilization and functional regulation of various immunologically important transcription factors. In addition, it contributes to chromatin decondensation and the formation of microtubules within the karyokinetic spindle [208], stabilizes the DNA double helix, and facilitates the transformation of a single DNA strand into a double strand [209]. Genome integrity disorders, inefficient enzymatic DNA repair mechanisms, and the loss of mechanisms controlling DNA function associated with zinc deficiency may lead to an increased risk of cancer initiation and progression [153]. Many proteins that excise damaged bases or nucleotides have a zinc finger domain and are therefore zinc-dependent, e.g., the p53 suppressor protein and AP (Apurinic/apyrimidinic) endonuclease [210]. The enzymes most essential to replication processes have also been shown to be zinc metalloenzymes, e.g., DNA and RNA polymerase, thymidine kinase, and replication protein A (RPA). RPA performs its function by altering the redox potential within the cell, owing to the presence of one of three subunits containing zinc fingers. Another example is the retinoic acid receptor (RAR), whose DNA-binding domain contains two zinc fingers that can release zinc ions under pro-oxidative conditions [211]. The most important protein containing zinc fingers and participating in repair processes involving nucleotide excision is XPA: human xeroderma pigmentosum group A C4-type zinc-finger protein. XPA has no catalytic activity, but its fragment containing zinc fingers plays a key role in recognizing and binding single-stranded DNA regions that have been destroyed as a result of pathological factors destabilizing the DNA helix [210]. Another function of XPA is recruiting repair proteins to the damaged region, including replicative protein A (RPA) [210]. The importance of zinc for the genome is confirmed by its involvement in numerous processes associated with the expression and stabilization of genes, as well as its key role in cell growth, cell division, and programmed death.

### 5.2. Zinc and Histone Deacetylases (HDACs)

The deacetylation of histones catalyzed by histone deacetylases (HDACs) results in the closer binding of histones to DNA and thus the inhibition of transcription processes [212]. HDACs have been divided into four classes [213]. Class I HDACs occur mainly in the cell nucleus, where they are involved in the regulation of the cell cycle, affecting the capacity of cancer cells for mitotic division. The only representative of HDAC class IV is HDAC11, which also occurs in the cell nucleus. HDAC2 is expressed only in certain tissues and, depending on the state of phosphorylation and binding to chaperones, they are able to move between the nucleus and the cytoplasm and prevent apoptosis. Class I, II, and IV enzymes belong to the family of classical histone deacetylases and contain a Zn^+2^ ion in the catalytic center. Class III HDACs are sirtuins (SIRT: silent information regulators), which are functionally and structurally completely different from the previous classes and require the presence of a cofactor, i.e., the oxidized form of nicotinamide adenine dinucleotide (NAD^+^), to function correctly [214]. In the majority of cancer cell types, the level of histone acetylation decreases due to the overexpression of HDACs, resulting in the abnormal transcriptional silencing of many genes [215]. A histone H4 anomaly often occurs as well, involving the loss of one acetyl residue at the Lys16 position and the triple methylation of Lys20. This type of change is considered to be a marker of the early stages of the neoplastic process [216]. However, it is still unknown which deacetylases are the most important for initiating and maintaining the metabolic pathways leading to tumor development. Nevertheless, new compounds have been created known as histone deacetylase inhibitors (HDIs), which block the activity of HDACs by interacting with their catalytic domain. This results in an increase in the level of histone acetylation, the formation of chromatin with a more open configuration, and the restoration of the expression of abnormally silenced genes that are essential for the functioning of the cell. HDIs can inhibit the cell cycle through various mechanisms. These compounds modulate the chromatin structure, leading to changes in the expression of a large number of genes influencing signaling pathways, the inhibition of the cell cycle and angiogenesis, or the induction of apoptosis in cancer cells. Currently, many types of HDIs are at the stage of clinical trials, being used in monotherapy or in combination with other cytostatics. To date, over 15 HDI compounds have been accepted as potential anti-cancer drugs, and 11 of these show a dependence on zinc ions [217]. The role of histone deacetylases, presently here in brief, indicates that zinc ions are closely linked to both the enzymes themselves and inhibitors of their action.

## 6. Zinc and Apoptosis and Autophagy

Apoptosis can be a response to irreparable DNA damage, and in germline cells, it represents the last chance to eliminate a hereditary genetic defect transferred by the spermatozoon or ovum. When apoptosis takes place in somatic cells with DNA damage, it is the last line of defense against the threat of neoplastic transformation. Thus far, zinc has been shown to have multi-directional activity in the initiation or inhibition of apoptosis through changes in its intracellular and extracellular concentration. By taking part in the regulation of apoptosis—the main mechanism of cell death in the body—zinc ensures the removal and destruction of mutant or damaged forms that can potentially lead to cancer [218].

An elevated Zn concentration inside the mitochondria causes impairment of the permeability of the mitochondrial membranes and the release of cytochrome C, thereby inhibiting respiratory chain reactions. This is followed by a surge in oxygen free radicals with a role in initiating apoptosis, exceeding the detoxifying capacity of the cell. Cytochrome C binds in the cytoplasm to the protein Apaf 1 (apoptotic protease activating factor 1) and to activated caspase 9, which triggers a cascade of biochemical changes resulting in irreversible structural changes in the cell. In addition to the schematically presented mechanism of proapoptotic action, many reports emphasize the opposite, anti-apoptotic effect of zinc ions. Supplementation to correct zinc deficiencies has been shown to prevent apoptosis induced by various factors, whereas a decreased level of zinc ions may intensify cell death in the apoptosis mechanism [219]. Zn generally contributes to the regulation of this main mechanism of cell death in the body, and ensures the removal and destruction of mutant or damaged forms. Deregulation of this process occurs in the pathogenesis of many diseases, including cancer [218,220]. In the cytoplasm of the dying cell, cysteine proteases of the ICE (interleukin-1-beta converting enzyme) family, called caspases (cysteine-dependent aspartate-directed proteases) are activated [188,221,222,223]. When the apoptosis process is activated, they undergo transformation from proenzymes into active forms affecting numerous intracellular substrates [224]. The active caspases activate more procaspases, leading to a cascade of reactions. This results in the destruction of structural proteins, e.g., cytoskeleton proteins, and enzymes such as poly (ADP-ribose) polymerase (PARP), which is responsible for DNA repair. The Rb protein regulating the cell cycle and MDM2 (MDM two binding protein) is cut as well. MDM2 blocks the ability of p53 to activate the genes associated with cell repair or apoptosis, and also leads to its degradation by ubiquitination. It also inhibits the suppressor properties of the pRb protein by releasing the E2F1 transcription factor, which induces the S-phase of DNA synthesis [223]. In addition, the signal transmitted by caspases leads to the induction of transcription factors, such as AP-1 and NF-κB. The structure and metabolic functions of the cell are disturbed, which leads to its death [224]. An appropriate concentration of zinc may be a factor that keeps caspase-3 in the form of a proenzyme, and its deficiency becomes a switch activating procaspase-3 [188,222,225]. Thus, a deficiency of zinc, a strong antioxidant, undoubtedly greatly weakens the cell, making it susceptible to free radicals released from the mitochondria. The accompanying lipid peroxidation and protein oxidation may additionally increase the activation of procaspase-3 [221].

It should be stressed that properly regulated apoptosis also maintains the balance in the immune system [226]. When cytotoxic T lymphocytes, whose action is dependent on zinc, are produced during disease, they cause apoptosis in cells that present fragments of foreign proteins on their surface, e.g., viral proteins. Then, the apoptotic signal is induced by extracellular signals associated with membrane receptors (FAS–FAS ligand) [62,227]. Apoptosis can also be induced in tumor cells by inhibiting their growth with tumor necrosis factor (TNF), which binds to the appropriate receptor on the surface of cancer cells and causes their elimination.

Suppression of the immune response after eradication of an infection leads to the death of a vast number of lymphocytes. This is followed by the elimination of autoreactive lymphocytes and limitation of the excessive expansion of lymphocytes responding to foreign antigens. In this context, both an increase and a decrease in apoptosis can lead to abnormalities and the accumulation of immune cells during inflammatory diseases, such as asthma [228].

Autophagy is the second type of programmed cell death [151,229]. Endogenous Zn seems to be indispensable for the induction of autophagy under oxidative stress conditions. After the start of this process, the level of Zn in the cell nucleus and the cytosol increases. It has been demonstrated that Zn chelation can inhibit the activity of lysosomes and decrease their passage through cell membranes, thereby preventing the release of acid hydrolases that break down proteins, nucleic acids, carbohydrates, and fats, leading to the self-digestion of the cell. Studies have also shown that zinc is a mediator of tamoxifen-induced autophagy in breast cancer cells [218].

In addition, the level of zinc can be modified by the microenvironment created in the tumor. Mast cells, which are present in large quantities in the altered tissue, release granules containing a significant amount of zinc. In addition, numerous cytokines and growth factors present in cancer tissue induce the expression of zinc transporters [230].

## 7. Zinc and the Psyche (NDMA Receptors)

Symptoms not directly related to oncological disease but often accompanying it include increased feelings of sadness and anxiety, low mood, a loss of interest in pleasures, reduced appetite, changes in body weight and sleep patterns, concentration disorders, and reduced cognitive abilities [231]. Zinc is important in the functioning of not only the immune system but also the central nervous system [232]. It is a significant element of antioxidant mechanisms that condition the integrity of the blood–brain barrier and protect against the harmful effects of oxidative stress. Zinc ions affect the redox status of brain cells, mainly due to its ability to inhibit N-methyl-D-aspartate (NMDA) receptors that influence the level of intracellular calcium and thereby regulate the activity of enzymes such as NADPH oxidase and nitric oxide synthase, which are a source of reactive oxygen species in many human tissues [233,234,235]. A lack of zinc in the blood is often linked to affective disorders, especially depression [236,237]. The pathophysiology and symptoms of depression are associated with increased glutamatergic transmission mediated by NMDA receptors and with an imbalance between pro- and anti-apoptotic factors. NMDA receptors are the target for the action of zinc, which functions as their modulator and inhibitor [238,239]. An activation of NMDA receptors requires, besides the presence of glutamic acid, glycine—or the closely related D-serine—to interact with it [238]. Zinc inhibits the flow of ions in NMDA receptors, reducing the affinity of glycine to the receptor. This amino acid is an essential factor in the NMDA receptor stimulation process. Without it, the flow of ions will not take place, regardless of the potency and quantity of circulating substances stimulating these receptors. Zinc is released from the glutamatergic neurons together with glutamate, allowing it to modulate its activity. This is one of the main antidepressant mechanisms of zinc.

## 8. Conclusions

The data cited indicate that zinc is an essential trace element for the activation or structural stabilization of a great number of enzymes and transcription factors as well as the immune and antioxidant response, apoptosis, and mental health. Supplementation and an optimal intake of zinc restore the normal immune response and reduce the risk of infection. However, the optimal immunostimulatory dose of zinc has not been determined. At the same time, it has been demonstrated that an excess amount of zinc can be dangerous due to its immunosuppressive effect. Knowledge of the dual effect of zinc is needed to evaluate its beneficial and negative effects in the prevention and treatment of cancer.
